# Statistical mediation by sleep quality in the association between menopausal symptoms and depressive symptoms in peri- and postmenopausal women: a multicenter cross-sectional study

**DOI:** 10.3389/fendo.2026.1915371

**Published:** 2026-09-08

**Authors:** Zhiling Guo, Dongjian Yang, Xiaoyi Wang, Shenglan Zuo, Danqi Liu, Mengfan Song, Furui Jin

**Affiliations:** 1 The International Peace Maternity and Child Health Hospital, School of Medicine, Shanghai Jiao Tong University, Shanghai, China; 2 Shanghai Key Laboratory of Embryo Original Diseases, Shanghai, China

**Keywords:** menopausal symptoms, sleep quality, depressive symptoms, statistical mediation, cross-sectional study

## Abstract

**Objective:**

This study aimed to examine the associations among menopausal symptoms, depressive symptoms, and sleep quality in peri- and postmenopausal women, with particular attention to the indirect statistical association through sleep quality.

**Methods:**

A multicenter cross-sectional study including 2,330 peri- and postmenopausal women was conducted. Menopausal symptoms were assessed using the Kupperman Menopausal Index (KMI), depressive symptoms using the Patient Health Questionnaire-9 (PHQ-9), and sleep quality using the Pittsburgh Sleep Quality Index (PSQI). Multivariable logistic regression and bootstrap-based statistical mediation models were applied to quantify associations and indirect effects.

**Results:**

Depressive symptoms (PHQ-9 ≥5) were observed in 34.0% of participants. Increasing severity of menopausal symptoms was significantly associated with both depressive symptoms and poor sleep quality (P<0.001). Poor sleep quality (PSQI >5) was strongly associated with depressive symptoms (odds ratio [OR]=4.48, 95% confidence interval [CI]: 3.72-5.40). Statistical mediation analysis indicated significant indirect associations through sleep quality, with proportions statistically mediated ranging from 17.08% to 63.39% and the largest proportion observed for hot flashes and sweating.

**Conclusion:**

Sleep quality statistically accounted for part of the observed association between menopausal symptoms and depressive symptoms. These findings support incorporating sleep assessment into menopause care and provide a rationale for prospective studies evaluating temporal relationships and the potential value of sleep-focused interventions.

## 1 Introduction

Menopause is a major endocrine transition in women’s life course and is characterized by declining ovarian function and fluctuating or reduced estrogen levels (1–3). These endocrine changes are accompanied by a broad range of symptoms, including vasomotor complaints, somatic discomfort, sleep disturbance, and mood-related manifestations (1, 3, 4). In addition to affecting quality of life, symptom burden during the menopausal transition is increasingly recognized as a contributor to long-term physical and mental health outcomes (5–7).

Perimenopause is not a fixed chronological-age interval. Under the Stages of Reproductive Aging Workshop +10 (STRAW + 10) framework, it begins with the early menopausal transition, marked by persistent menstrual-cycle variability, and extends through the first 12 months after the final menstrual period (FMP); its duration varies substantially, with a median of approximately four years (8). For mutually exclusive study categories, we operationally used “perimenopausal” for women in the early or late menopausal transition and “postmenopausal” for women with at least 12 months of amenorrhea after the FMP.

Depressive symptoms are of particular clinical concern during peri- and postmenopause. Midlife women experiencing moderate-to-severe menopausal symptoms are more likely to report low mood, irritability, anxiety, and impaired psychosocial functioning (9–12). Sleep disturbance is also highly prevalent during the menopausal transition and may be closely associated with both menopausal symptom burden and mood dysregulation (13–16). Sleep disorders in women are influenced by reproductive stage and gynecologic factors, and the menopausal transition is a particularly vulnerable period in which hormonal changes, vasomotor symptoms, and psychosocial factors may jointly disrupt sleep (17). Vasomotor symptoms, especially hot flashes and sweating, may trigger repeated nocturnal arousals and sleep fragmentation, thereby amplifying daytime fatigue, emotional distress, and depressive symptoms (13–16).

From a pathophysiological perspective, endocrine aging may affect the hypothalamic-pituitary-ovarian axis and downstream neurobiological systems involved in sleep and mood regulation (1, 18). Reduced estrogen exposure has been associated with altered serotonergic and GABAergic signaling, changes in thermoregulation, and increased vulnerability to stress-system activation (9, 19). Accordingly, sleep quality may be considered a clinically relevant correlate within the observed association between menopausal symptoms and depressive symptoms, rather than evidence of a confirmed intermediary mechanism (19).

Against this background, we conducted a multicenter cross-sectional study of women aged 40–60 years recruited from four medical institutions in Shanghai, China, to evaluate the associations among menopausal symptoms, sleep quality, and depressive symptoms and to quantify the indirect statistical association through sleep quality. We hypothesized that: (1) greater menopausal symptom burden would be associated with depressive symptoms; (2) sleep quality would be associated with both menopausal symptoms and depressive symptoms; and (3) an indirect statistical association through sleep quality would be detectable between menopausal symptoms and depressive symptoms (20–22).

## 2 Materials and methods

### 2.1 Study design and participants

This multicenter cross-sectional study was conducted between March 1, 2021 and December 13, 2023 at four medical institutions in Shanghai, China: the International Peace Maternity and Child Health Hospital affiliated with Shanghai Jiao Tong University School of Medicine, Xidu Community Health Service Center in Fengxian District, Tinglin Hospital in Jinshan District, and the Qingpu Branch of Zhongshan Hospital.

Eligible participants were women aged 40–60 years who were classified as being in the early or late menopausal transition or postmenopause according to STRAW + 10 and who voluntarily completed the study survey. The 40-60-year range was selected to capture the common midlife window of menopausal transition and early postmenopause. Reproductive stage was assigned from menstrual-pattern criteria rather than chronological age; therefore, women in their early forties who met transition criteria were not considered abnormal solely because of age. Natural menopause before age 45 is conventionally considered early, whereas loss of ovarian function before age 40 is considered premature (8, 23). Women were excluded if they had used menopausal hormone therapy within the previous 3 months, had severe cardiovascular, immune, or hematologic disease, or had a diagnosed psychiatric disorder that prevented survey completion. Women with self-reported hysterectomy or bilateral oophorectomy before natural menopause were excluded because reproductive stage could not be classified using menstrual bleeding criteria.

The reporting of results follows the Strengthening the Reporting of Observational Studies in Epidemiology (STROBE) guidelines.

**Figure 1:**
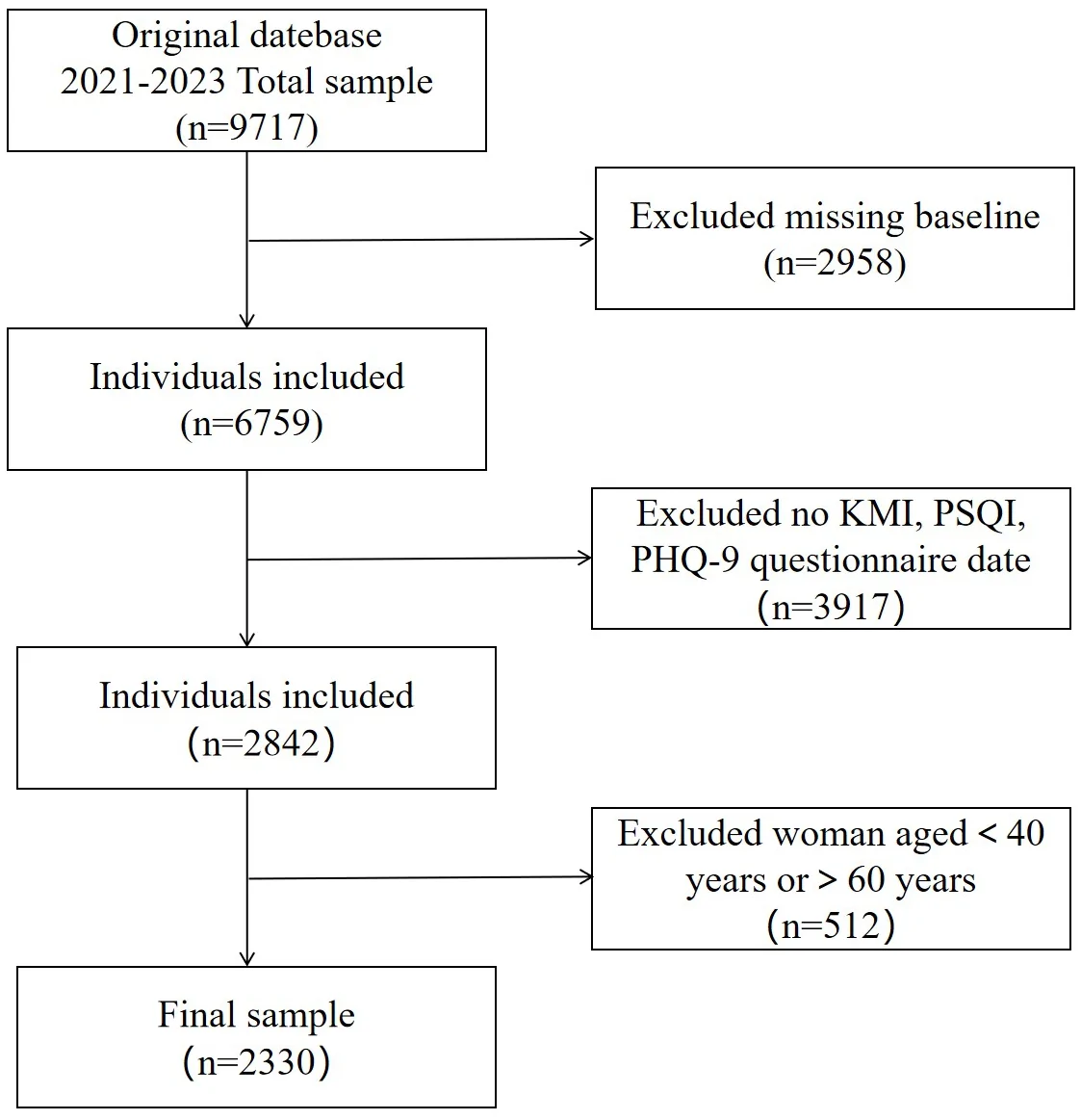
Flowchart of the study.

A total of 9,717 women were initially identified from the database. Missingness was evaluated separately for each questionnaire: 1,607 participants had incomplete KMI data, 5,079 had incomplete PHQ-9 data, and 4,484 had incomplete PSQI data. Questionnaire missingness reflected either non-completion of the entire instrument or omission of one or more individual items. Because some participants had incomplete data for more than one questionnaire, these categories were not mutually exclusive and should not be summed. After the sequential exclusions shown in **Figure 1** for missing baseline information, incomplete questionnaire data, and age criteria, 2,330 participants with complete baseline, KMI, PHQ-9, and PSQI data were included in the complete-case analysis.

### 2.2 Menopausal status

Menopausal status was classified according to the STRAW + 10 criteria (8). Early menopausal transition was defined by a persistent difference of at least 7 days in the length of consecutive menstrual cycles, and late menopausal transition by an interval of amenorrhea lasting at least 60 days. Postmenopause was defined after 12 consecutive months of amenorrhea following the FMP. Although clinical perimenopause may extend through the first 12 months after the FMP, the analytic categories were made mutually exclusive: early and late menopausal transition were grouped as perimenopause, and women after 12 months of amenorrhea were classified as postmenopausal.

### 2.3 Data collection and measures

Menopausal symptoms were assessed using the Kupperman Menopausal Index (KMI) (24), which evaluates common menopausal symptoms including hot flashes and sweating, paresthesia, vertigo, arthralgia/myalgia, headache, palpitations, decreased libido, and urinary tract infection. Symptom severity was categorized as none, light, or moderate/severe. Total KMI scores were categorized as normal (≤6), light (7–15), and moderate/severe (≥16).

Depressive symptoms were assessed using the Patient Health Questionnaire-9 (PHQ-9) (25). The PHQ-9 was used as a symptom severity measure rather than a diagnostic instrument. Because the study aimed to capture the broader burden of depressive symptoms during the menopausal transition, including early and subthreshold symptoms, participants with PHQ-9 scores ≥5 were classified as having at least mild depressive symptoms, whereas those with scores <5 were classified as having no or minimal depressive symptoms. In the original validation study, scores of 5 and 10 represented mild and moderate symptom levels, respectively, while a cutoff of ≥10 was more closely associated with probable major depression. Accordingly, the outcome in this study should be interpreted as depressive symptoms and not as a clinical diagnosis of depression.

Sleep quality was assessed using the Pittsburgh Sleep Quality Index (PSQI) (26), with a total score >5 indicating poor sleep quality. No disorder-specific validated screening instruments for restless legs syndrome, obstructive sleep apnea, or insomnia were administered; therefore, the PSQI findings reflect global self-reported sleep quality and not diagnoses of specific sleep disorders. The survey did not include a comprehensive inventory of prescription or over-the-counter medications that could affect sleep, cognition, or mood; menopausal hormone therapy was the only medication-related exclusion specified *a priori*.

### 2.4 Statistical analysis

Continuous variables were summarized as mean (standard deviation), and categorical variables as number (percentage). Baseline characteristics were compared using independent-samples t-tests for continuous variables and chi-square tests for categorical variables.

Multiple logistic regression models were used to estimate odds ratios (ORs) and 95% confidence intervals (CIs) for: (1) the association between menopausal symptoms and depressive symptoms; (2) the association between poor sleep quality and depressive symptoms; and (3) the association between menopausal symptoms and poor sleep quality. Three models were reported: an unadjusted model, Model I adjusted for age, BMI, marital status, and financial income, and Model II additionally adjusted for menopausal status, abortion history, and reproductive history.

A bootstrap-based statistical mediation analysis was conducted to estimate the indirect association through sleep quality in the relationship between menopausal symptoms and depressive symptoms. Because the exposure, mediator, and outcome were measured cross-sectionally, the indirect effect was interpreted as statistical mediation and not as evidence of a causal or temporally ordered pathway. The symptom-specific analyses were hypothesis-driven, and no formal adjustment for multiple comparisons was applied; *P* values should therefore be interpreted as nominal and considered together with effect sizes and 95% confidence intervals. Statistical significance was defined as a two-sided P value <0.05. Analyses were performed using SPSS version 24.0.

## 3 Results

### 3.1 Baseline characteristics

Among the 2,330 participants, 1,538 (66.0%) were classified as having no depressive symptoms and 792 (34.0%) as having depressive symptoms. Compared with the non-depressive symptoms group, women with depressive symptoms were more likely to be overweight or obese, less likely to be married, more likely to be in the late menopausal transition or postmenopausal stage, and more likely to report a history of abortion. They also had substantially higher prevalence and severity across all evaluated menopausal symptoms and a markedly higher prevalence of poor sleep quality (63.6% vs. 27.7%) (**Table 1**).

**Table 1:** Baseline characteristics of the study participants.

Characteristics	All participants (n=2330)	PHQ<5 (n=1538)	PHQ ≥ 5 (n=792)	*P*-value
Age, years, n (%)				0.124
40-44	473 (20.3)	293 (19.1)	180 (22.7)	
45-49	898 (38.5)	590 (38.4)	308 (38.9)	
50-54	731 (31.4)	499 (32.4)	232 (29.3)	
55-60	228 (9.8)	156 (10.1)	72 (9.1)	
BMI, kg/m^2^, n (%)				0.007
< 18.5	76 (3.3)	59(3.8)	17(2.1)	
18.5~23.9	1651(70.9)	1106(71.9)	545(68.8)	
≥ 24	603(25.9)	373(24.3)	230(29.0)	
Marital status, n (%)				0.024
Married	2096(90.0)	1399(91.0)	697(88.0)	
Single or other	234(10.0)	139(9.0)	95(12.0)	
Financial income, RMB, n (%)				0.227
< 5000	835 (35.8)	535(34.8)	300(37.9)	
5000~10000	1,277 (54.8)	851(55.3)	426(53.8)	
> 10000	218(9.4)	152(9.9)	66(8.3)	
Menopausal status, n (%)				0.027
Early menopausal transition	653 (28.0)	458 (29.8)	195 (24.6)	
Late menopausal transition	968 (41.5)	618 (40.2)	350 (44.2)	
Postmenopause	709 (30.4)	462 (30.0)	247 (31.2)	
Abortion, n (%)				0.001
No	615 (26.4)	439 (28.5)	176 (22.2)	
Yes	1715 (73.6)	1099 (71.5)	616 (77.8)	
Reproductive history, n (%)				0.094
No	122 (5.2)	72 (4.7)	50(6.3)	
Yes	2208(94.8)	1466 (95.3)	742 (93.7)	
Hot flashes and sweating, n (%)				< 0.001
No	936 (40.2)	709 (46.1)	227 (28.7)	
Light	805 (34.5)	484(31.5)	321(40.5)	
Moderate/severe	589(25.3)	345(22.4)	244(30.8)	
Paresthesia, n (%)				< 0.001
No	1235 (53.0)	930 (60.5)	305 (38.5)	
Light	378 (16.2)	222 (14.4)	156 (19.7)	
Moderate/severe	717 (30.8)	386 (25.1)	331 (41.8)	
Vertigo, n (%)				< 0.001
No	1132 (48.6)	881 (57.3)	251 (31.7)	
Light	995 (42.7)	574 (37.3)	421 (53.2)	
Moderate/severe	203 (8.7)	83 (5.4)	120 (15.2)	
Arthralgia and myalgia, n (%)				< 0.001
No	868 (37.3)	662 (43.0)	206 (26.0)	
Light	910 (39.1)	598 (38.9)	312 (39.4)	
Moderate/severe	552 (23.7)	278 (18.1)	274 (34.6)	
Headache, n (%)				< 0.001
No	1022 (43.9)	796 (51.8)	226 (28.5)	
Light	1025 (44.0)	626 (40.7)	399 (50.4)	
Moderate/severe	283 (12.1)	116 (7.5)	167 (21.1)	
Palpitation, n (%)				< 0.001
No	992 (42.6)	805 (52.3)	187 (23.6)	
Light	1073 (46.1)	640 (41.6)	433 (54.7)	
Moderate/severe	265 (11.4)	93 (6.0)	172 (21.7)	
Decreased libido, n (%)				< 0.001
No	800 (34.3)	617 (40.1)	183 (23.1)	
Light	1182 (50.7)	739 (48)	443 (55.9)	
Moderate/severe	348 (14.9)	182 (11.8)	166 (21.0)	
Urinary tract infection, n (%)				< 0.001
No	1576 (67.6)	1112 (72.3)	464 (58.6)	
Light	567 (24.3)	335 (21.8)	232 (29.3)	
Moderate/severe	187 (8.0)	91 (5.9)	96 (12.1)	
KMI total scores, n (%)				< 0.001
Normal	313 (13.4)	301 (19.6)	12 (1.5)	
Light	750 (32.2)	592 (38.5)	158 (19.9)	
Moderate/severe	1267 (54.4)	645 (41.9)	622(78.5)	
PSQI, n (%)				< 0.001
≤5	1400 (60.1)	1112 (72.3)	288 (36.4)	
>5	930 (39.9)	426 (27.7)	504 (63.6)	

BMI, body mass index; PHQ-9, Patient Health Questionnaire-9; KMI, Kupperman Menopausal Index; PSQI, Pittsburgh Sleep Quality Index; RMB, renminbi.

### 3.2 Association between menopausal symptoms and depressive symptoms

In the fully adjusted model, increasing severity of each menopausal symptom was associated with higher odds of depressive symptoms, with clear dose-response trends across symptom categories. The strongest associations were observed for palpitations, headache, and vertigo. Elevated risks were also evident for hot flashes and sweating, paresthesia, arthralgia/myalgia, decreased libido, and urinary tract infection. (**Table 2**).

**Table 2:** Association between symptoms of menopause and depressive symptoms in the multiple regression model.

Menopausal symptom/severity	Unadjusted Model ^a^		Adjusted model I ^b^		Adjusted model II ^c^	
	OR (95% CI)	*P*-value	OR (95% CI)	*P*-value	OR (95% CI)	*P*-value
Hot flashes and sweating						
No	1(Ref.)		1(Ref.)		1(Ref.)	
Light	2.07 (1.69~2.54)	< 0.001	2.08(1.70~2.56)	< 0.001	2.03 (1.65~2.51)	< 0.001
Moderate/severe	2.21 (1.77~2.76)	< 0.001	2.19(1.75~2.74)	< 0.001	2.10(1.66~2.65)	< 0.001
* P* trend		< 0.001		< 0.001		< 0.001
Paresthesia						
No	1(Ref.)		1(Ref.)		1(Ref.)	
Light	2.14 (1.68~2.73)	< 0.001	2.13(1.67~2.72)	< 0.001	2.12 (1.66~2.7)	< 0.001
Moderate/severe	2.61 (2.15~3.18)	< 0.001	2.61(2.14~3.18)	< 0.001	2.52 (2.06~3.08)	< 0.001
* P* trend		< 0.001		< 0.001		< 0.001
Vertigo						
No	1(Ref.)		1(Ref.)		1(Ref.)	
Light	2.57 (2.13~3.11)	< 0.001	2.60(2.15~3.14)	< 0.001	2.61 (2.16~3.16)	< 0.001
Moderate/severe	5.07 (3.71~6.94)	< 0.001	5.23(3.82~7.18)	< 0.001	5.10 (3.71~7.01)	< 0.001
* P* trend		< 0.001		< 0.001		< 0.001
Arthralgia and myalgia						
No	1(Ref.)		1(Ref.)		1(Ref.)	
Light	1.68 (1.36~2.06)	< 0.001	1.67(1.36~2.06)	< 0.001	1.69 (1.37~2.08)	< 0.001
Moderate/severe	3.17 (2.52~3.98)	< 0.001	3.16(2.51~3.98)	< 0.001	3.12 (2.46~3.94)	< 0.001
* P* trend		< 0.001		< 0.001		< 0.001
Headache						
No	1(Ref.)		1(Ref.)		1(Ref.)	
Light	2.24 (1.85~2.73)	< 0.001	2.27(1.87~2.76)	< 0.001	2.31 (1.90~2.81)	< 0.001
Moderate/severe	5.07 (3.84~6.70)	< 0.001	5.22(3.94~6.92)	< 0.001	5.15 (3.88~6.83)	< 0.001
* P* trend		< 0.001		< 0.001		< 0.001
Palpitation						
No	1(Ref.)		1(Ref.)		1(Ref.)	
Light	2.91 (2.38~3.56)	< 0.001	2.92(2.39~3.58)	< 0.001	2.87 (2.35~3.52)	< 0.001
Moderate/severe	7.96 (5.91~10.73)	< 0.001	8.12(6.01~10.97)	< 0.001	7.99 (5.89~10.83)	< 0.001
* P* trend		< 0.001		< 0.001		< 0.001
Decreased libido						
No	1(Ref.)		1(Ref.)		1(Ref.)	
Light	2.02 (1.65~2.48)	< 0.001	1.98(1.62~2.43)	< 0.001	1.92 (1.56~2.37)	< 0.001
Moderate/severe	3.08 (2.35~4.02)	< 0.001	3.06(2.34~4.00)	< 0.001	3.10 (2.34~4.12)	< 0.001
* P* trend		< 0.001		< 0.001		< 0.001
Urinary tract infection						
No	1(Ref.)		1(Ref.)		1(Ref.)	
Light	1.66 (1.36~2.03)	< 0.001	1.68(1.38~2.06)	< 0.001	1.65 (1.35~2.02)	< 0.001
Moderate/severe	2.53 (1.86~3.44)	< 0.001	2.61(1.91~3.56)	< 0.001	2.52 (1.84~3.45)	< 0.001
* P* trend		< 0.001		< 0.001		< 0.001

OR, odds ratio; CI, confidence interval; Ref., reference category; BMI, body mass index.

^a^ Unadjusted model: no covariates were adjusted. ^b^Adjusted model I: adjusted for age, BMI, marital status, and financial income. ^c^Adjusted model II: adjusted for age, BMI, marital status, financial income, menopausal status, abortion history, and reproductive history.

### 3.3 Association between sleep quality and depressive symptoms

Poor sleep quality was strongly associated with depressive symptoms. In the fully adjusted model, participants with PSQI >5 had 4.48-fold higher odds of depressive symptoms (95% CI: 3.72-5.40) compared with those with PSQI ≤5 (**Table 3**).

**Table 3:** Association between sleep quality and depressive symptoms in the multiple regression model.

Measure	Category	Unadjusted Model ^a^		Adjusted model I ^b^		Adjusted model II ^c^	*P*-value
		OR (95% CI)	*P*-value	OR (95% CI)	*P*-value	OR (95% CI)	
PSQI	≤5	1(Ref.)		1(Ref.)		1(Ref.)	
	>5	4.57 (3.8~5.48)	< 0.001	4.55(3.78~5.47)	< 0.001	4.48 (3.72~5.40)	< 0.001

PSQI, Pittsburgh Sleep Quality Index; OR, odds ratio; CI, confidence interval; Ref., reference category; BMI, body mass index.

^a^ Unadjusted model: no covariates were adjusted. ^b^Adjusted model I: adjusted for age, BMI, marital status, and financial income. ^c^Adjusted model II: adjusted for age, BMI, marital status, financial income, menopausal status, abortion history, and reproductive history.

### 3.4 Association between menopausal symptoms and poor sleep quality

Increasing severity of menopausal symptoms was also associated with increased odds of poor sleep quality. Particularly strong associations were observed for palpitations, decreased libido, paresthesia, and hot flashes and sweating, indicating that symptom burden is closely related to sleep disruption in peri- and postmenopausal women (**Table 4**).

**Table 4:** Association between symptoms of menopause and poor sleep quality in the multiple regression model.

Menopausal symptom/severity	Unadjusted Model ^a^		Adjusted model I ^b^		Adjusted model II ^c^	P-value
	OR (95% CI)	P-value	OR (95% CI)	P-value	OR (95% CI)	
Hot flashes and sweating						
No	1(Ref.)		1(Ref.)		1(Ref.)	
Light	2.93 (2.39~3.60)	< 0.001	2.94 (2.39~3.61)	< 0.001	2.67 (2.16~3.29)	< 0.001
Moderate/severe	4.28 (3.43~5.35)	< 0.001	4.28 (3.42~5.35)	< 0.001	3.68 (2.92~4.63)	< 0.001
* P* trend		< 0.001		< 0.001	< 0.001	
Paresthesia						
No	1(Ref.)		1(Ref.)		1(Ref.)	
Light	2.13 (1.68~2.71)	< 0.001	2.13 (1.68~2.71)	< 0.001	2.09 (1.64~2.66)	< 0.001
Moderate/severe	4.27 (3.51~5.20)	< 0.001	4.28 (3.52~5.21)	< 0.001	3.89 (3.19~4.75)	< 0.001
* P* trend		< 0.001		< 0.001		< 0.001
Vertigo						
No	1(Ref.)		1(Ref.)		1(Ref.)	
Light	1.49 (1.25~1.78)	< 0.001	1.5 (1.25~1.78)	< 0.001	1.48 (1.24~1.77)	< 0.001
Moderate/severe	2.85 (2.1~3.87)	< 0.001	2.87 (2.11~3.9)	< 0.001	2.71 (1.98~3.7)	< 0.001
* P* trend		< 0.001		< 0.001		< 0.001
Arthralgia and myalgia						
No	1(Ref.)		1(Ref.)		1(Ref.)	
Light	1.37 (1.13~1.67)	0.001	1.37 (1.13~1.67)	0.001	1.35 (1.11~1.65)	0.003
Moderate/severe	2.59 (2.07~3.22)	< 0.001	2.60 (2.09~3.25)	< 0.001	2.35 (1.87~2.95)	< 0.001
* P* trend		< 0.001		< 0.001		< 0.001
Headache						
No	1(Ref.)		1(Ref.)		1(Ref.)	
Light	1.47 (1.23~1.76)	< 0.001	1.48 (1.23~1.77)	< 0.001	1.53 (1.27~1.83)	< 0.001
Moderate/severe	3.16 (2.41~4.15)	< 0.001	3.21 (2.44~4.21)	< 0.001	3.13 (2.37~4.13)	< 0.001
* P* trend		< 0.001		< 0.001		< 0.001
Palpitation						
No	1(Ref.)		1(Ref.)		1(Ref.)	
Light	2.30 (1.91~2.77)	< 0.001	2.30 (1.91~2.77)	< 0.001	2.19 (1.81~2.64)	< 0.001
Moderate/severe	5.64 (4.21~7.56)	< 0.001	5.66 (4.22~7.59)	< 0.001	5.23 (3.88~7.04)	< 0.001
* P* trend		< 0.001		< 0.001		< 0.001
Decreased libido						
No	1(Ref.)		1(Ref.)		1(Ref.)	
Light	2.74 (2.24~3.34)	< 0.001	2.72 (2.22~3.32)	< 0.001	2.43 (1.98~2.98)	< 0.001
Moderate/severe	4.50 (3.44~5.89)	< 0.001	4.49 (3.43~5.88)	< 0.001	3.94 (2.97~5.21)	< 0.001
* P* trend		< 0.001		< 0.001		< 0.001
Urinary tract infection						
No	1(Ref.)		1(Ref.)		1(Ref.)	
Light	1.15 (0.95~1.4)	0.152	1.16 (0.95~1.41)	0.143	1.11 (0.91~1.36)	0.303
Moderate/severe	2.23 (1.64~3.03)	< 0.001	2.25 (1.65~3.07)	< 0.001	2.05 (1.50~2.82)	< 0.001
* P* trend		< 0.001		< 0.001		< 0.001

OR, odds ratio; CI, confidence interval; Ref., reference category; BMI, body mass index.

^a^ Unadjusted model: no covariates were adjusted. ^b^Adjusted model I: adjusted for age, BMI, marital status, and financial income. ^c^Adjusted model II: adjusted for age, BMI, marital status, financial income, menopausal status, abortion history, and reproductive history.

### 3.5 Statistical mediation analysis

**Figure 2:**
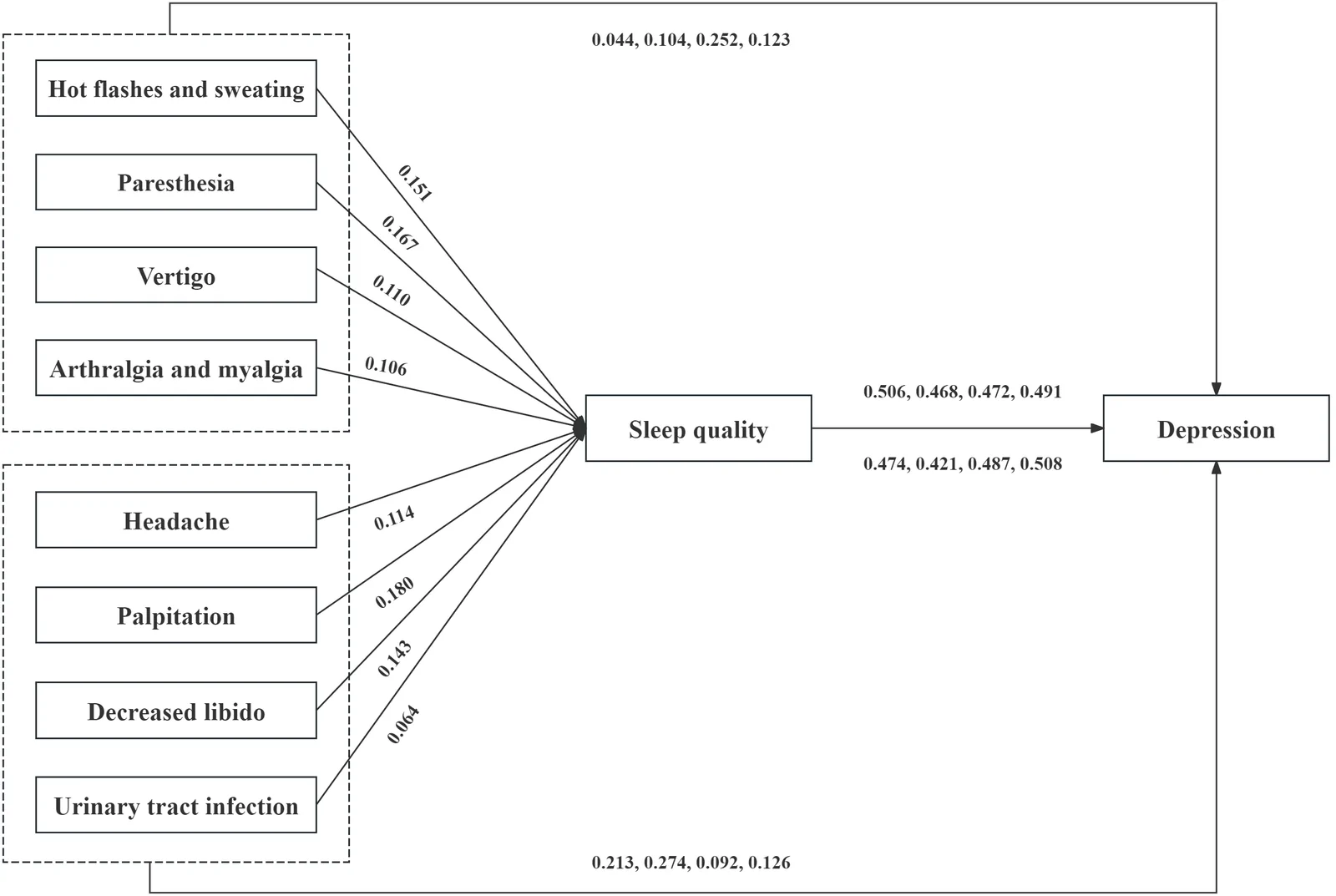
Cross-sectional statistical mediation models of menopausal symptoms, sleep quality, and depressive symptoms. Separate symptom-specific models are shown. Values on the symptom-to-sleep and sleep-to-depressive-symptom arrows are the estimated a- and b-path coefficients; values on the outer paths are direct effects (c′). Upper and lower coefficient sequences correspond to the symptoms listed in the upper and lower boxes, respectively. Estimates are from the bootstrap models in **Table 5**.

Bootstrap-based statistical mediation analysis identified significant indirect associations through sleep quality (**Table 5**). The proportions statistically mediated ranged from 17.08% for vertigo to 63.39% for hot flashes and sweating. These estimates describe the statistical decomposition of cross-sectional associations and do not establish a temporal or causal pathway. A summary of the statistical mediation model is presented in **Figure 2**.

**Table 5:** Statistical mediation model of menopausal symptoms, sleep quality, and depressive symptoms.

Menopausal symptom	Overall effect (95% CI)	*P*-value	Indirect effect (95% CI)	*P*-value	Direct effect (95% CI)	*P*-value	Indirect/total (%)
Hot flashes and sweating	0.121(0.086~0.155)	< 0.001	0.077(0.072~0.104)	< 0.001	0.044(0.010~0.078)	< 0.001	63.39%
Paresthesia	0.183(0.150~0.216)	< 0.001	0.078(0.078~0.115)	< 0.001	0.104(0.072~0.137)	< 0.001	42.81%
Vertigo	0.304(0.262~0.347)	< 0.001	0.052(0.032~0.066)	< 0.001	0.252(0.212~0.293)	< 0.001	17.08%
Arthralgia and myalgia	0.175(0.139~0.212)	< 0.001	0.052(0.042~0.071)	< 0.001	0.123(0.088~0.158)	< 0.001	29.83%
Headache	0.267(0.227~0.307)	< 0.001	0.054(0.036~0.067)	< 0.001	0.213(0.174~0.252)	< 0.001	20.21%
Palpitation	0.350(0.310~0.390)	< 0.001	0.076(0.062~0.090)	< 0.001	0.274(0.234~0.314)	< 0.001	21.72%
Decreased libido	0.162(0.127~0.196)	< 0.001	0.070(0.064~0.093)	< 0.001	0.092(0.058~0.126)	< 0.001	43.09%
Urinary tract infection	0.158(0.118~0.199)	< 0.001	0.033(0.017~0.049)	< 0.001	0.126(0.088~0.164)	< 0.001	20.54%

CI, confidence interval; PSQI, Pittsburgh Sleep Quality Index. Overall, indirect, and direct effects are regression coefficients from separate symptom-specific bootstrap mediation models; indirect/total (%) is the ratio of the indirect effect to the overall effect.

## 4 Discussion

This multicenter study investigated the relationships among menopausal symptoms, depressive symptoms, and sleep quality in peri- and postmenopausal women, with particular emphasis on the indirect statistical association through sleep quality. The main finding was that greater menopausal symptom severity was associated with a higher burden of depressive symptoms and that sleep quality statistically accounted for part of this observed association. This pattern was present across vasomotor and non-vasomotor symptom domains, including hot flashes and sweating, vertigo, decreased libido, and urinary tract-related symptoms (27, 28). These findings identify sleep quality as an important correlate within the co-occurrence of menopausal and depressive symptoms, without establishing that sleep disturbance causally transmits an effect.

Compared with previous studies, our results provide further evidence supporting the close association between menopausal symptoms and depressive symptoms. However, most prior research has focused primarily on pairwise or direct associations (29, 30). Our findings extend the current literature by quantifying indirect statistical associations through sleep quality. In the context of cross-sectional data, this result indicates that part of the observed association is shared with sleep quality; it does not demonstrate an intermediate physiological or behavioral pathway. This adds new evidence supporting a more integrative understanding of menopausal symptom clustering, sleep, and mood regulation.

From a mechanistic perspective, the observed associations may be interpreted in light of, but do not directly demonstrate, a neuroendocrine aging framework. Menopausal transition is characterized by dysregulation of the hypothalamic–pituitary–ovarian (HPO) axis and progressive estrogen decline (31, 32), with downstream effects on neurotransmitter systems involved in sleep and mood regulation (33, 34). Estrogen modulates serotonergic signaling by regulating tryptophan hydroxylase and serotonin receptors (5-HT1A/5-HT2A), which are essential for sleep stability and emotional regulation (35, 36). Its decline is associated with reduced serotonergic tone, REM sleep instability, increased nocturnal awakenings, and greater mood vulnerability (36–38). In parallel, reduced estrogen and neurosteroids such as allopregnanolone may impair GABA__A_ receptor-mediated inhibition, decreasing sleep stability and promoting NREM sleep fragmentation (39–41). Sleep disturbances may also be associated with mood dysregulation through activation of the hypothalamic–pituitary–adrenal (HPA) axis, inflammatory pathways, and impaired emotional recovery (42). These mechanisms provide biological plausibility for the co-occurrence of sleep disturbance and mood symptoms. However, because endocrine biomarkers and temporal data were not collected, the present results cannot establish that sleep quality is a neuroendocrine intermediary between hormonal decline and affective regulation.

In addition to biological mechanisms, psychosocial factors may further contribute to the observed associations. Women in midlife often experience concurrent stressors, including occupational burden, family role transitions, and concerns related to aging and health perception (43). These psychosocial stressors may interact with physiological endocrine changes, exacerbating sleep disturbance and emotional dysregulation. Notably, subjective sleep quality appears to be more strongly associated with depressive symptoms than objective sleep parameters, suggesting that cognitive and emotional appraisal plays an important role in shaping sleep-related outcomes (43, 44). Furthermore, anxiety and depressive symptoms frequently coexist during menopause and exhibit bidirectional interactions with sleep disturbance, potentially forming a self-reinforcing cycle that exacerbates symptom severity (43, 44).

Clinically, these findings support the importance of integrating sleep assessment into the routine evaluation of menopausal women. From a gynecologic perspective, sleep complaints during the menopausal transition should be assessed systematically, with attention to symptom context, potential coexisting sleep disorders, and individualized management considerations (17). The Pittsburgh Sleep Quality Index (PSQI) may help identify women with poor sleep quality; depressive symptoms require separate assessment using an appropriate instrument such as the PHQ-9 and, when indicated, clinical evaluation. Sleep-related indicators may nevertheless provide useful contextual information when evaluating women with moderate-to-severe menopausal symptoms (45). Because the present findings are cross-sectional, the effectiveness of sleep-focused interventions for reducing subsequent depressive symptom burden should be evaluated in prospective and interventional studies.

This study has several strengths, including its multicenter design, relatively large sample size, and comprehensive assessment of menopausal symptoms, sleep quality, and depressive symptoms. Nevertheless, several limitations should be acknowledged. First, due to the cross-sectional design, temporal ordering and causal relationships cannot be inferred, and the observed indirect effects should be interpreted as statistical rather than causal mediation. Second, self-reported measures may introduce recall and reporting bias. Third, the complete-case analysis excluded participants with incomplete baseline or questionnaire data; because questionnaire missingness was substantial and overlapping, selection bias cannot be excluded. Fourth, the lack of endocrine biomarkers such as estradiol, follicle-stimulating hormone, and luteinizing hormone limits direct mechanistic evaluation of the proposed neuroendocrine context. Fifth, the study did not include validated disorder-specific screening for restless legs syndrome, obstructive sleep apnea, or insomnia, and detailed use of prescription and over-the-counter medications with potential effects on sleep, cognition, or mood was not systematically recorded. Residual confounding from unrecognized sleep disorders or medication exposure is therefore possible.

Future studies should further examine whether reproductive stage modifies the relationships among menopausal symptoms, sleep quality, and depressive symptoms. Stage-stratified analyses with formal interaction testing may clarify whether the magnitude of these associations and indirect statistical effects differs between perimenopausal and postmenopausal women. More importantly, longitudinal studies with repeated assessments and precise information on time before and after the final menstrual period are needed to determine how these symptom-sleep-mood relationships evolve as women progress through the menopausal transition.

## 5 Conclusion

Sleep quality statistically accounted for part of the observed association between menopausal symptoms and depressive symptoms in peri- and postmenopausal women. Because the data were cross-sectional, this indirect association should not be interpreted as evidence of a causal pathway. These findings support the incorporation of sleep assessment into routine menopause care and provide a rationale for prospective studies examining temporal endocrine, sleep, and mood relationships and evaluating sleep-focused interventions.

## Data Availability

The original contributions presented in the study are included in the article/supplementary material, further inquiries can be directed to the corresponding author/s.
